# Antidiabetic Drugs in Breast Cancer Patients

**DOI:** 10.3390/cancers16020299

**Published:** 2024-01-10

**Authors:** Wojciech Garczorz, Agnieszka Kosowska, Tomasz Francuz

**Affiliations:** Department of Biochemistry, Faculty of Medical Sciences in Katowice, Medical University of Silesia, Medyków 18, 40-055 Katowice, Poland; akosowska@sum.edu.pl (A.K.); tfrancuz@sum.edu.pl (T.F.)

**Keywords:** diabetes, breast cancer, antidiabetic drugs, metastasis, insulin, metformin, incretin agonists, gliflozins, sulfonylureas, thiazolidinediones

## Abstract

**Simple Summary:**

Diabetes affects patients with breast cancer. Women with diabetes and the comorbidities related to diabetes tend to receive less aggressive breast cancer treatments primarily because of the potential side effects of the treatment. Managing proper antidiabetic treatment should affect the patient’s breast cancer prognosis in patients with both conditions. This review explores the link between diabetes and an elevated risk of breast cancer, highlighting the global prevalence of these conditions. It delves into the impact of antihyperglycemic therapy, including insulin, metformin, and novel groups: incretins and SGLT-2 inhibitors, on the prognosis of breast cancer in diabetic patients. The review also underscores the increased incidence and mortality rates of breast cancer in individuals with diabetes, along with the role of obesity and its connection to the metastasis process in those undergoing antidiabetic treatment. The role of obesity as a confounding variable is confirmed, highlighting the importance of considering obesity during hypoglycemic therapy selection in breast cancer patients.

**Abstract:**

Diabetes is one of the leading chronic conditions worldwide, and breast cancer is the most prevalent cancer in women worldwide. The linkage between diabetes and its ability to increase the risk of breast cancer should always be analyzed in patients. This review focuses on the impact of antihyperglycemic therapy in breast cancer patients. Patients with diabetes have a higher risk of developing cancer than the general population. Moreover, diabetes patients have a higher incidence and mortality of breast cancer. In this review, we describe the influence of antidiabetic drugs from insulin and metformin to the current and emerging therapies, incretins and SGLT-2 inhibitors, on breast cancer prognosis. We also emphasize the role of obesity and the metastasis process in breast cancer patients who are treated with antidiabetic drugs.

## 1. Introduction

Diabetes (DM) and cancer are diseases that interact with each other, cause life-altering morbidity, and high rates of mortality. Type 1 diabetes is based on autoimmune destruction of pancreatic β cells, usually leading to absolute insulin deficiency. Therefore, insulin therapy is necessary from the beginning of the treatment. Approximately 6% of patients suffer from type 1 diabetes. The most common is type 2 diabetes (T2DM), which covers more than 90% of diabetic patients. The two main reasons for T2DM are impairment of insulin secretion and resistance to the action of insulin (insulin resistance), which may be caused by both genetic factors and obesity. Type 2 diabetes is a chronic, lifelong metabolic disease and is one of the most common metabolic abnormalities in Western populations, which makes it an increasing global public health concern [1]. The latest data presented by International Diabetes Federation in 2021 reveals that 537 million adults around the world suffer from diabetes, and by 2045, there will be 783 million diabetics [2]. Both types of diabetes increase a person’s risk of serious complications. The metabolic and hormonal consequences of diabetes, and its treatment, might also affect the risk of malignancy. Most studies of cancer risks in patients with diabetes are related to DM. Common risk factors for both cancer and diabetes are aging, sex, obesity, physical inactivity, diet, alcohol, and smoking [3]. The main goal of T2DM treatment is to achieve the optimal glycemic control, with a target hemoglobin A1C (HbA1C) level <7% and the prevention of micro- and macrovascular complications [4].

**Figure 1:**
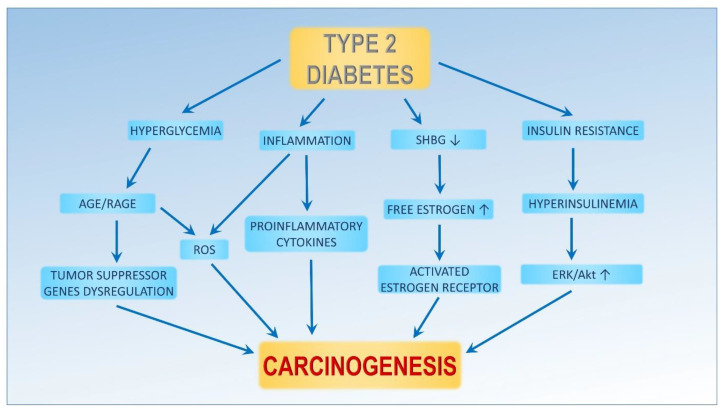
Diabetes ameliorates carcinogenesis. An increasing production of sex hormones and decreasing sex hormone binding globulin (SHBG) production causes high plasma-free estrogen concentrations, which activate the estrogen receptor (ER). Hyperglycemia and inflammation are related to diabetes. Both processes lead to an increase in reactive oxygen species (ROS) and advanced glycation end products (AGE). Insulin resistance leads to high plasma insulin concentrations, which activate ERK and the Akt pathways. Activation of these pathways can lead to proliferation, invasiveness, angiogenesis, and decreased apoptosis.

Carcinogenesis is a complex process that starts with a normal cell that undergoes multiple genetic changes before it fully becomes neoplastic, invades, and metastasizes. The oncogenesis undergoes distinct phases, initiation, promotion, and progression, indicating the development of a more aggressive phenotype in the promoted cells. Any factors influencing these stages may be linked to the onset of cancer incidents. Breast cancer (BCa) is the most common malignant tumor in women. BCa is a malignant tumor originating from the epithelium of the ducts or lobules of the mammary gland, and therefore, they are classified as adenocarcinomas. It can occur in both women and men; although, it is much more common in women. The most common types of BCa are invasive ductal carcinoma and invasive lobular carcinoma. Less common types of BCa are inflammatory breast cancer and Paget’s disease of the breast which primarily affects about 1 to 4 percent of patients. Diabetes can impact the neoplastic process through various mechanisms, such as hyperinsulinemia, hyperglycemia, or chronic inflammation (Figure 1) [5,6].

## 2. Epidemiology—Diabetes and Breast Cancer

Epidemiological studies have confirmed that DM is associated with an increased risk of cancer, including cancers of the breast, colon, liver, pancreas, and kidney [7]. Interestingly, diabetes is also associated with a decreased risk of prostate cancer, probably due to a lower testosterone level [8]. Diabetes and breast cancer (BCa) represent prevalent chronic conditions in the female population. Current estimates indicate that 16% of individuals diagnosed with breast cancer also have diabetes [7]. Breast cancer stands as one of the most frequently encountered neoplasms and a significant contributor to cancer-related mortality in women, accounting for 685,000 deaths in the year 2020 [9]. The concurrent presence of diabetes and breast cancer may impact treatment strategies, thereby exerting a potential negative influence on overall prognosis. An increasing number of incidences of breast cancer has been observed in Western countries, mainly because of a sedentary lifestyle and unhealthy diet. It has been observed that women that have diabetes have a worse survival prognosis after a breast cancer diagnosis compared to women without diabetes. Different etiologies might lead to the development of more aggressive breast cancer subtypes. A meta-analysis of 23 studies found that diabetes is associated with an increased mortality hazard ratio (HR = 1.41, 95% CI: 1.28–1.55) in individuals with cancer, including breast cancer [10]. Today, screening programs have been implemented in many countries for some cancers, including breast cancer. It was reported that comorbidities and advanced-stage diagnosis are associated with poorer cancer outcomes. Such associations were analyzed by Boakye et al. in a study that included 8,069,397 cancer patients who were suffering from lung, breast, colorectal, and prostate cancer. They found that the Charlson Comorbidity Index (CCI) score was positively associated with stages III or IV of breast cancer. Regarding specific comorbidities, diabetes was positively associated with an advanced-stage diagnosis (OR 1.17, 95% CI: 1.09–1.26) [11].

In a recent meta-analysis by Xiong et al., an investigation was carried out to assess the correlation between diabetes and the risk of developing breast cancer (BCa). The findings indicated that diabetes was linked to an overall heightened risk of BCa, with a relative risk (RR) of 1.20 and a 95% confidence interval (CI) of 1.11–1.29 [12]. Furthermore, the analysis revealed that postmenopausal women exhibited an increased susceptibility to developing BCa, with an RR of 1.12 and a 95% CI of 1.07–1.17. Conversely, no significant association was observed between diabetes and the risk of BCa among premenopausal women, with an RR of 0.95 and a 95% CI of 0.85–1.05.

Metastasis and the invasion of cancer cells into new tissue and other organs are the major problems in cancer treatment [13]. The tumor microenvironment is a heterogeneous mixture of tumor cells and endogenous host stroma that changes during the cancer progression [14,15]. Stromal cells, including fibroblasts, endothelial cells, pericytes, adipocytes, MSCs, and immune cells, play a multitask role in the development of the tumor microenvironment, metastasis, immune infiltration, and inflammation, as well as to the resistance to chemotherapeutic agents [16]. Additionally, the upregulation of plasminogen activator inhibitor-1 (PAI-1) in breast cancer is associated with an adverse prognosis. The induction of signal transducer and activator of transcription proteins (STAT) signaling through cytokines such as interleukin-6 (IL-6) is recognized to augment cancer cell proliferation, enhance survival, and promote invasion. Concurrently, this signaling pathway has been documented to suppress the host’s anti-tumor immune response [17]. Each of these factors can exert a pivotal influence on the progression of cancer.

### Obesity and Breast Cancer Risk

Beyond the direct impacts of insulin, obesity may contribute to the activation of alternative pathways, potentially leading to malignant progression. Adipose tissue, recognized as an active endocrine organ, generates various bioactive molecules such as interleukin-6 (IL-6), monocyte chemoattractant protein, plasminogen activator inhibitor-1, adiponectin, leptin, and tumor necrosis factor alpha (TNFα). These adipose-derived substances play integral roles in mediating physiological processes and may influence cancer-related pathways, thereby contributing to the progression of malignancy [18]. Recent investigations have presented findings suggesting that metabolic health, as opposed to body mass index (BMI), may serve as a more accurate predictor of breast cancer risk [19]. In clinical trials, a body mass index (BMI) greater than 30 is commonly used as the threshold to define obesity.

A recent meta-analysis exploring the correlation between obesity and breast cancer revealed a protective role of obesity in pre-menopausal European women (odds ratio [OR] = 0.88; 95% confidence interval [CI]: 0.79–0.98; I2 = 44.8%). However, this protective effect was not observed in post-menopausal women, where obesity was associated with an increased risk (OR = 1.26, 95% CI: 1.19–1.34) [20]. Yifan Lu et al. conducted a subgroup analysis within the meta-analysis, focusing on 20 studies. Among the seven studies that adjusted breast cancer risk for BMI, an elevated risk was identified (RR = 1.22, 95% CI: 1.15–1.30) [21]. This trend aligns with findings from Boyle et al. and Hardefeldt et al., who, in their subgroup analyses with BMI adjustments, reported an increased risk of breast cancer associated with obesity (RR = 1.16, 95% CI: 1.08–1.24 and RR = 1.12, 95% CI: 1.04–1.21, respectively) [22,23]. Adipose tissue, which is abundant in obese individuals, is metabolically active and produces hormones and cytokines that can influence breast cancer development. The promotion of breast cancer growth in the context of obesity involves the regulation of pro-proliferative and anti-apoptotic transcriptional programs in cancer cells, as well as effects on microenvironment oxidative stress. Targeting dysfunctional obese breast adipose tissue through weight reduction or pharmacological approaches has shown promise in decreasing breast cancer risk [24].

Novel antidiabetic medications have been found to influence weight reduction. Recent meta-analyses have indicated an anti-obesity effect of GLP-1 receptor agonists, including liraglutide, exenatide, and semaglutide, particularly in obese individuals without diabetes. In comparison to a placebo, the weighted mean difference (WMD) in mass reduction was significant (WMD = −5.39, 95% CI: −6.82–−3.96), and when compared to metformin, the WMD was −5.46 (95% CI: −5.87–−5.05) [25]. Notably, semaglutide demonstrated the most substantial anti-obesity effect, resulting in a reduction in body mass index (BMI). However, it is important to note that this was associated with a higher incidence of gastrointestinal adverse events, such as nausea and vomiting, compared to the placebo.

## 3. Hyperglycemic Action of Anti-Cancer Drugs

Several anti-cancer agents may lead to hyperglycemia. Such side-effects may also worsen the clinical status of treated patients. As indicated in the phase I study involving patients with breast cancer (BCa) and substantiated by larger phase II studies, the majority of first-line drugs are not associated with hyperglycemia. However, temsirolimus and everolimus which are approved for the treatment of BCa rates of hyperglycemia (all grades) ranged from 7% to 93% [26]. The drugs within this group share a common mechanism of action, as they function as inhibitors of the mammalian target of rapamycin (mTOR). The deregulation of numerous components of the mTOR pathway, including phosphoinositide 3-kinase (PI3K), phosphatase and tensin homolog (PTEN), and protein kinase B (AKT) has been related to many cancers, including BCa. These drugs bind to both mTOR and an important coactivator, FKBP12, leading to a change in the 3D shape of the proteins. It prevents the binding of RAPTOR, a protein required for the activation of downstream pathways. These pathways regulate proliferation and survival as well as glucose homeostasis. Therefore, its inhibition may lead to hyperglycemia.

## 4. Antidiabetic Drugs and Its Role in Breast Cancer

### 4.1. Insulin

Experimental models have emphasized the influence of increased levels of insulin and insulin-like growth factors (IGF) on enhanced tumorigenesis. Insulin and IGF are peptides that are crucial for glucose homeostasis, cell proliferation, metabolism, differentiation, and apoptosis.

The insulin/IGF signaling system primarily involves three ligands—IGF-1, IGF-2, and insulin—which can interact with at least six receptors: the type 1 IGF receptor (IGF-1R), insulin receptor A (IR-A), insulin receptor B (IR-B), hybrid receptors of IGF with IR-A, hybrid receptors of IGF with IR-B, and hybrid receptors of IR-A with IR-B [27]. Insulin and its analogs, both of which elevate circulating insulin levels, have been documented to heighten the risk of pancreatic and colorectal cancers [28]. Insulin and IGFs activate metabolic and mitogenic signaling pathways, with insulin additionally downregulating insulin-like growth factor-binding protein (IGF-BP) and sex hormone-binding globulin (SHBG). This downregulation contributes to IGF and steroid hormone-dependent breast cancers [29]. Elevated circulating insulin levels also induce angiogenesis and foster tumor growth through activated mitogenic signaling mechanisms [30].

Most concerns are about the long-acting analogs (insulin glargine, detemir, and degludec). To date, most studies on the insulin analogs and cancer have had a short follow-up (less than five years). A long-term evaluation of this connection is necessary [15]. Another reason is that hyperglycemia provides a nutrient-rich environment for rapidly dividing cancer cells, which have a higher metabolic rate activity than normal cells [16]. In a retrospective study that included 462 DM breast cancer patients and 1644 non-diabetic breast cancer patients, Mu et al. analyzed the influence of insulin treatment. The five-year disease-free survival (HR = 1.75, 95% CI: 1.37–2.25; *p* < 0.001) and overall survival (HR = 1.96, 95% CI: 1.44–2.66; *p* < 0.001) were diminished in patients with diabetes. The detailed results were presented following adjustments for factors including age, tumor size, histological grade, estrogen receptor, progesterone receptor, chemotherapy, and hormone therapy. In this study, the five-year risks of relapse (HR = 1.61; 95% CI: 1.07–2.41; *p* = 0.021) and mortality (HR = 1.68, 95% CI: 1.03–2.75; *p* = 0.038) revealed a significant increase in the insulin subgroup when compared to the non-insulin subgroup, even after considering all of the previously mentioned factors [31]. A potential weakness of this study was the choice of a non-diabetic group for comparison. Other observations and conclusions were drawn from a meta-analysis by Janghorbani et al. They showed that insulin treatment was associated with an increased risk of overall cancer (RR = 1.39, 95% CI: 1.14–1.70), but if the influence of insulin treatment specifically on breast, prostate, and hepatocellular cancer was considered, it was not associated, and the estimates of their effect were not statistically significant [29]. In the meta-analysis by Wang et al. on the impact of insulin use on the outcomes of diabetic breast cancer, eleven studies were included. Their results demonstrated a significant increase in the risk of breast cancer mortality amongst insulin users compared to non-users (HR = 1.33, 95% CI: 1.08–1.63; *p* = 0.007). Only four of the eleven studies assessed the impact of insulin treatment on a recurrence of the cancer. A meta-analysis of these studies indicated a statistically significant increased risk of breast cancer recurrence in insulin users vs. non-users (HR = 1.43, 95% CI: 1.13–1.80; *p* = 0.003) [32]. However, a contrary result was revealed by another study. An interesting systematic review and meta-analysis that evaluated the associations between diabetes medications and the risk of different cancers was recently published by Chen et al. A total of 92 studies involving 171 million participants were included in the systematic review. Based on the 15 studies that were included, a lower risk of breast cancer (RR = 0.90, 95% CI: 0.82–0.98) was found among insulin users compared to nonusers [33].

Insulin analogs exhibit varying affinities to IGF-1R and IR-A, potentially leading to increased mitogenic activity of these analogs. Earlier epidemiological studies have raised concerns, particularly regarding insulin glargine, suggesting a potential association with an elevated risk of cancer. Some evidence suggested that insulin glargine might pose a higher risk of cancer compared to human insulin. In a systematic review and meta-analysis by Bronsveld et al. focusing on insulin analogs and breast cancer risk, glargine was identified as the only clinically available insulin analog showing enhanced proliferative potential in breast cancer cell lines. However, the results of a meta-analysis, incorporating 13 epidemiological studies, failed to provide conclusive evidence linking insulin glargine treatment to an increased risk of breast cancer (HR = 1.04, 95% CI: 0.91–1.17; *p* = 0.49) when compared to the absence of glargine treatment in patients with diabetes mellitus [34].

**Figure 2:**
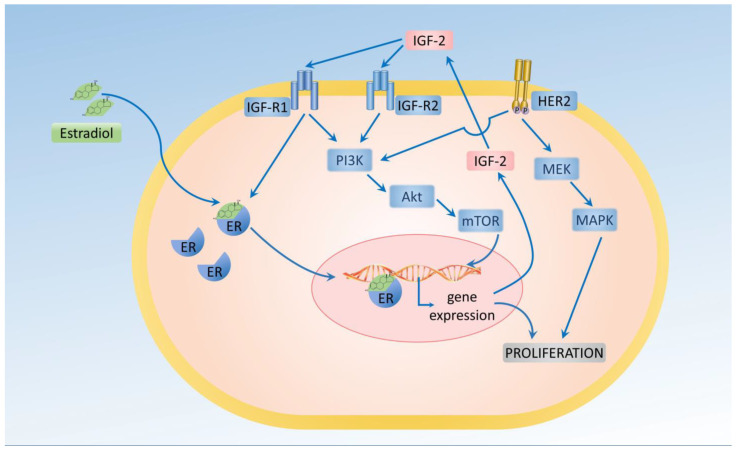
Estrogen (estradiol) stimulates the proliferation of breast cancer cells.

Hormonal control disruption, mainly including estrogens, is considered to play a crucial role in promoting the proliferation of neoplastic breast epithelium. Mammary gland epithelial cells express estrogen receptors (ERs) and progesterone receptors (PRs). In breast cancer, interruption of the estrogen receptor α (ER-α) function is an effective therapeutic strategy. Despite the clinical benefit of interrupting the ER-α function, the precise biological action of ER-α in breast tumors remains unelucidated. The estrogens that bind to its receptors regulate gene transcription, as well as having extra-nuclear effects on the activation of many signaling molecules, including MAPK and Akt. In vitro studies have demonstrated that ER can influence the membrane growth factor receptor signaling pathways involving IGF-1-R and EGF-R [35,36] (Figure 2).

Breast cancer cells can respond to insulin, especially in diabetic patients with hyperinsulinemia. Because of the homology, insulin and IGF-1 can interact with either IR or with IGF-IR. Hyperinsulinemia also leads to increased estrogen levels, which is crucial for tumor development. One of the latest meta-analyses by Drummond et al. confirmed that a higher IGF-1 level increased the risk of breast cancer (RR = 1.21, 95% CI: 1.10–1.31, I2= 0%). This finding was supported by the Mendelian randomization studies. The same manuscripts reported that IGFBP-3 did not affect breast cancer (RR = 0.97, 95% CI: 0.70–1.24, I2= 0.0%) [37].

### 4.2. Sulfonylureas

Sulfonylureas (SUs) have been used in the treatment of DM since the 1950s. The absorption of the therapeutic doses of SUs is reached within 30 min, with a peak insulinotropic response within two to three hours. Sulfonylureas are recommended for diabetic patients who are not overweight. Moreover, SUs should be used in patients in whom metformin is contraindicated or is not strong enough to obtain the appropriate level of glycemic control. The second generation of sulfonylureas (gliclazide, glipizide, glibenclamide, and glimepiride) is currently being used. Sulfonylureas stimulate insulin secretion by the pancreatic β-cells and decrease the hepatic clearance of insulin, which is observed after an increase in insulin secretion [38]. Sulfonylureas are usually well tolerated. Nevertheless, sulfonylureas have been associated with the highest risk of hypoglycemia among the oral glucose-lowering agents, especially in their long-acting forms [39].

In a meta-analysis of the observational studies by Sorrana et al., 1068 patients with breast cancer were included. While they reported that sulfonylureas did not affect the risk of cancer at any site including the breast, no specific data were provided [40]. Chen et al. conducted a retrospective cohort study using the Surveillance, Epidemiology and End-Results (SEER)-Medicare database. In the study, the authors included 16,397 women, aged 66–80 years, who had been diagnosed with stage I or II breast cancer. Time-dependent Cox proportional hazard models were used to estimate the hazard ratios. The use of sulfonylureas was associated with (HR = 1.49, 95% CI: 1.00–2.23) higher risks of death due to breast cancer. However, among the diabetic women who were being treated, the recurrence of the breast cancer or death did not differ between the users and non-users of sulfonylureas [41]. The more recent results of the Lawrence et al. study on 9221 women with breast cancer concluded that compared to women using metformin, the all-cause mortality hazard was higher among the patients who were using sulfonylurea (HR = 1.44, 95% CI: 1.06–1.94) [42]. Similar results were obtained in an analysis of two population-based studies in Shanghai. Six hundred thirty-three patients with breast cancer were included and the associations between the use of diabetes medication and survival were evaluated using time-dependent Cox proportional hazard models. The use of sulfonylureas was associated with a worse overall survival rate for breast cancer (HR = 2.87, 95% CI: 1.22–6.80) among the diabetic patients [43]. Despite the fact that sulfonylureas have been available for many years and are effective at a low cost, there is still a lack of good quality prospective studies on their impact on patients with cancer. In 2017, the ZODIAC-55 study was begun. It is a prospective study that investigates the differences among sulfonylureas in the risk for all cancers combined and site-specific cancers separately (i.e., breast, colorectal, prostate, bladder, and lung cancer) and the relationships between the use of individual sulfonylureas and the cancer risk [44]. There is no data from this study to date.

### 4.3. Thiazolidinediones

Thiazolidinediones (TZDs) are insulin-sensitizing peroxisome proliferator-activated receptor (PPARγ) agonists that modulate the expression and repression of specific genes without directly increasing insulin secretion. Currently available on the market, pioglitazone and rosiglitazone belong to this class of drugs. In vitro studies have demonstrated that PPARγ agonists, including TZDs, possess several anti-cancer properties, such as inducing apoptosis and inhibiting growth. Consequently, PPARγ agonists are currently being considered as a potential target for cancer therapy [45,46].

Despite the promising in vitro findings, studies on this matter have been inconsistent and limited. In vitro experiments with cancer cells have suggested that TZDs have the capacity to inhibit cancer cell growth, proliferation, and induce apoptosis [30]. A meta-analysis conducted by Du et al. on breast cancer risk among diabetic women found no significant association between the use of TZDs and the risk of breast cancer. In both randomized controlled trials (RCTs) (RR = 0.77; 95%; CI: 0.39–1.53, I2 = 26%) and case–control studies (OR = 0.99, 95% CI: 0.76–1.28, I2 = 31%), no significant associations between TZD use and breast cancer risk were observed [47]. In contrast, a more recent meta-analysis by Chen et al., incorporating six studies, reported that the use of thiazolidinediones was associated with a lower risk of breast cancer (RR = 0.87, 95% CI: 0.80–0.95) [33].

### 4.4. Metformin

Metformin, which is a biguanide family drug that is used in the treatment of diabetes, that reduces the glucose level and improves insulin sensitivity in peripheral tissues, may also have an anti-cancer effect [48]. The anticarcinogenic effects of metformin have been ascribed to several mechanisms such as the activation of the adenosine monophosphate-activated protein kinase AMPK/LKB1 pathway, the inhibition of protein synthesis, the inhibition of the unfolded protein response (UPR), the induction of apoptosis or cell cycle arrest of stem cells, or the rapid activation of immune response. The human tumor suppressor liver kinase B1 (LKB1), also known as serine/threonine kinase 11, directly phosphorylates and activates AMPK, which is a central metabolic sensor. AMPK regulates fat and glucose metabolism in specialized tissues, such as muscle, liver, and adipose tissue [49,50]. Epidemiological studies of patients using metformin showed that they had a lower cancer incidence and higher cancer survival rate [51,52]. Preclinical studies have also shown the anti-tumorigenic effect of metformin in breast, prostate, and colon cancer [53,54,55]. A recent retrospective cohort study of 3553 patients with breast cancer and DM showed significant survival differences among the non-diabetes group, metformin group, and insulin group; the five-year disease-free survival rate (DFS) was 85.8%, 96.1%, and 73.0%, and the five-year overall survival (OS) was 87.3%, 97.1%, and 73.3%, respectively. This provides a theoretical background for introducing metformin for the treatment of cancer and improving the overall survival rate [56].

Studies involving non-diabetic breast cancer patients treated with metformin are currently limited. In a meta-analysis of nine clinical trials, Farkhondeh et al. concluded that patients with operable breast and endometrial cancer undergoing metformin therapy showed no significant changes in investigated metabolic biomarkers, including homeostasis model assessment-estimated insulin resistance (HOMA-IR), body mass index (BMI), or fasting plasma sugar [57]. Due to the high heterogeneity of the included results, their findings could not confirm or reject the efficacy of metformin for patients with breast and endometrial cancer.

Metformin, beyond its primary anti-hyperglycemic actions, has demonstrated vascular protective properties and direct anti-cancer effects. These effects are attributed to the inhibition of hepatic gluconeogenesis, diminished insulin signaling through the suppression of the PI3K cellular response, and increased AMPK activity within cancer cells. Elevated AMPK activity leads to the downstream inhibition of PI3K/Akt/mTOR and Ras/RafMEK/ERK signaling pathways, suppressing protein synthesis and proliferation [58]. Furthermore, metformin reduces circulating estrogen levels, which have been associated with postmenopausal breast cancer development [59]. Metformin also mediates apoptosis by increasing oxidative stress after activating the AMPK and forkhead transcription factor 3 (FOXO3) protein [58].

Studies by Chlebowski et al. have indicated that diabetic patients taking metformin had a lower incidence of invasive breast cancer compared to those taking other antidiabetic medications such as sulfonylureas. Metformin administration in women with diabetes was associated with a reduced incidence of breast cancer (HR = 0.75, 95% CI: 0.57–0.99). This correlation extended to cancers positive for both estrogen and progesterone receptors and those negative for human epidermal growth factor receptor 2 (HER2) [60]. Data from the ALTTO Phase III Randomized Trial showed that HER2-positive diabetic breast cancer patients on metformin exhibited higher disease-free survival and overall survival rates compared to those not on metformin treatment [61]. Patients with diabetes not treated with metformin experienced lower rates of disease-free survival (DFS), distant disease-free survival (DDFS), and overall survival (OS). However, the positive impact of metformin was primarily observed in hormone-receptor-positive patients, suggesting potential improvement in prognosis associated with diabetes and insulin treatment in HER2-positive and hormone-receptor-positive breast cancer patients. In a meta-analysis by Yang et al., assessing the prognostic value of metformin in various cancers, including 31,031 breast cancer patients (3936 metformin users), metformin therapy demonstrated potential survival benefits compared to non-metformin users, including overall survival (HR = 0.77, 95% CI: 0.69–0.86) and progression-free survival (HR = 0.64, 95% CI: 0.44–0.91) [62]. However, biases were observed among the studies. On the contrary, data from the M.A.32 phase-three randomized study suggested that adding metformin to standard breast cancer treatment did not significantly improve disease-free survival or other breast cancer outcomes in patients without diabetes [63]. The possible reason for the discrepancy between ALTTO and M.A.32 studies was the presence of diabetes. All patients using metformin in the ALTTO study had both BCa and DM, but the M.A.32 study recruited BCa patients without DM.

Another meta-analysis evaluated the risk of breast cancer in women with diabetes associated with metformin use. This analysis, including 15 studies, found that for women with diabetes, the relative risk of breast cancer was 0.82 (95% CI: 0.60–1.12) for metformin users compared to non-metformin users [21]. However, this study did not confirm any clear advantage of metformin in lowering the risk of breast cancer.

In vitro studies have shown synergistic interactions between metformin and the EGFR tyrosine kinase inhibitor erlotinib in triple-negative breast cancer cell lines (TNBC) [64]. However, in a phase I clinical trial of erlotinib and metformin in metastatic TNBC patients who had received at least one prior line of therapy, no significant clinical benefits were observed among the small number of patients included in the study [65].

In the MYME clinical trial (phase II), assessing the effectiveness of metformin in first-line chemotherapy in nondiabetic patients with HER2-negative metastatic breast cancer, no advantages were observed in terms of progression-free survival (PFS) or overall survival (OS) in the metformin-treated group. However, metformin demonstrated a positive impact on insulin sensitization and exhibited significant preventive effects on severe neutropenia induced by chemotherapy [24].

### 4.5. Incretin Agonists

Gastrointestinal cells secrete about 30 peptides that have an endocrine activity [66]. Incretins are a group of metabolic hormones that work as glucose-lowering agents. The gastrointestinal tract secretes incretin hormones in response to food intake, which has many systemic effects on the body including the glucose-dependent stimulation of insulin secretion by the pancreatic beta cells. Two main incretin hormones, glucagon-like peptide 1 (GLP-1) and glucose-dependent insulinotropic peptide also known as gastric inhibitory peptide (GIP) have been identified. GLP-1 is derived from the L-cells of the distal small intestine and subsequently large bowel, but GIP is released from K-cells of the proximal small intestine [67,68]. Many hormones have been thought to contribute to the incretin system.

Animal studies and in vitro studies have shown that increased incretin leads to β-cell proliferation and the inhibition of its apoptosis. Based on these studies, there were some concerns about the potential carcinogenesis of β-cells. However, this effect does not occur in people with type 2 diabetes. The incretin effect is significantly impaired in people with type 2 diabetes, mainly due to the reduced secretion of GLP-1 [69,70]. The biological effects that are exerted by the GLP-1 receptor (GLP-1R) in these individuals are not disturbed, which makes therapy that is aimed at substituting GLP-1 reasonable. Natural GLP-1 therapy has significant limitations related to its rapid degradation by dipeptidyl peptidase-4 (DPP-4). As a result, practical therapy uses of the GLP-1 analogs, which are resistant to DPP-4 such as exenatide or liraglutide, are characterized by a subcutaneous biological activity of up to five to seven hours [71,72], unless they are administered in a slow-releasing form.

Reports indicate that the use of GLP-1 receptor agonists (GLP-1RAs) for the treatment of obesity and diabetes does not appear to elevate the risk of breast neoplasms. In a comprehensive analysis by Piccoli et al., covering 52 clinical trials with 50 reporting breast cancer events and 11 reporting benign breast neoplasms, a meta-analysis involving 48,267 patients treated with GLP-1RAs revealed that 130 individuals developed breast cancer, compared to 107 cases in the control group (RR = 0.98, 95% CI: 0.76–1.26). Additionally, the risk of benign breast neoplasms did not differ significantly between the analyzed groups (RR = 0.99, 95% CI: 0.48–2.01) [73].

Another population-based observational study conducted by Caparrotta et al. included 200,148 participants with 396,457 person years of follow-up. The study analyzed the exposure to the GLP-1RA class, specifically exenatide and liraglutide, in relation to various health outcomes. For safety outcomes, liraglutide exposure was not associated with an increased risk of breast cancer, with a point estimate range (PER) of 0.90–1.51. It is worth noting that the other outcomes had insufficient data for firm conclusions and warrant further exploration [74].

### 4.6. Dipeptidyl Peptidase-4 Inhibitors

**Figure 3:**
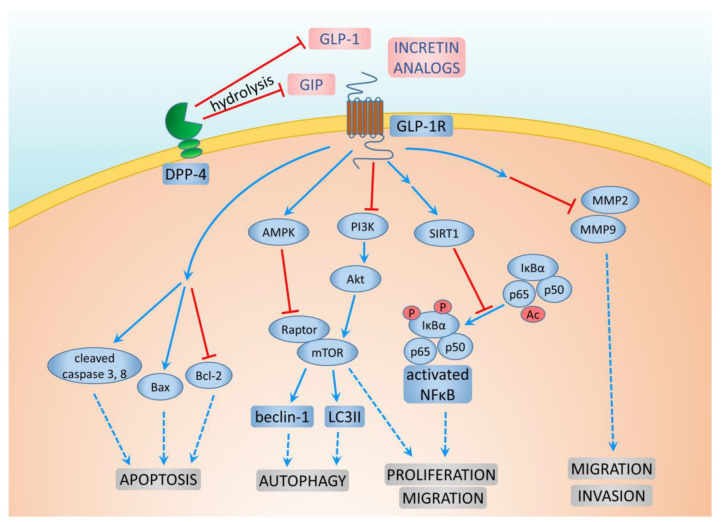
Mechanisms of GLP-1 and DPP-4 action on breast cancer cells.

Dipeptidyl peptidase (DPP) belongs to the family of serine proteases (S9B), which are responsible for regulating a variety of important biological processes. The best-studied member of this family is DPP-4, which is responsible for the hydrolysis of GLP-1 and GIP. DPP-4 is a transmembrane protein that has enzymatic activity, which is also involved in the signal transduction process. DPP-4 has a number of functions in the immune system, the central nervous system, and endocrine and participates in cell adhesion, tumor development, and the regulation of the inflammatory processes [75] (Figure 3). DPP-4 is widely expressed on the surface of endothelial and epithelial cells. This enzyme is normally bound to the cell membrane, but in certain conditions, it may be cleaved and released into the bloodstream. Therefore, active DPP-4 can be observed in the blood serum. DPP-4, which is associated with the cell membrane, plays an important role in the formation of complexes with adenosine deaminase, regardless of their enzymatic functions, and is involved in signal transduction into a cell [76]. The inhibition of DPP-4 can also have a beneficial effect on the cardiovascular system [71]. In a meta-analysis conducted by Noh et al., no significant metastatic risk was observed with DPP-4 inhibitors in various primary cancers, including breast cancer, when compared to no antidiabetic therapy among diabetic cancer patients [77].

Additionally, metformin use, either alone or in combination with DPP-4 inhibitors, was associated with a lower risk of new-onset metastasis in diabetic patients with concomitant cancer, with hazard ratios of 0.84 (95% CI: 0.79–0.90) and 0.87 (95% CI: 0.80–0.95), respectively [78]. Kawakita et al. reported that DPP-4 inhibitor treatment accelerated mammary cancer metastasis by inducing the epithelial–mesenchymal transition (EMT) through the CXCL12/CXCR4/mTOR axis. However, metformin, known for its inhibition of the mTOR signaling pathway, was suggested to counteract the unfavorable effects of DPP-4 inhibitors on breast cancer metastasis by suppressing mTOR, indicating its potential clinical relevance [78].

Another intriguing mechanism was elucidated by Wang et al. in an animal model. In vivo data revealed a dual role of the nuclear factor E2–related factor 2 (NRF2) in cancer. In normal cells, NRF2 acted as a tumor suppressor by preventing reactive oxygen species (ROS)-induced DNA damage, while in cancer cells, it functioned as an oncogene by promoting cancer cell survival in an ROS-rich tumor microenvironment. Testing two DPP-4 inhibitors, saxagliptin and sitagliptin, the researchers found that these inhibitors induced prolonged activation of the NRF2-mediated antioxidant response, leading to overexpression of metastasis-associated proteins, increased cancer cell migration, and enhanced metastasis in xenograft mouse models. They suggested caution in administering antioxidants that activate NRF2 signaling in cancer patients, such as diabetic patients with cancer. Moreover, NRF2 was proposed as a potential biomarker and therapeutic target for tumor metastasis [79].

Previously, there were concerns about the influence of DPP-4 inhibition on carcinogenesis, particularly in the pancreas and thyroid. The meta-analysis by Overbeek et al. revealed that for both pancreatic cancer (RR = 0.55, 95% CI: 0.29–1.03) and thyroid cancer (HR = 1.61, 95% CI: 0.99–2.62), the risk was not changed significantly. They reported that the influence of DPP-4 inhibitors on the risk of breast cancer was not significant in the results of randomized controlled trials (RR = 0.74, 95% CI: 0.36–1.52), but that in the results of observational analysis, such risk is decreased (HR = 0.76, 95% CI: 0.60–0.96) [80]. The average follow-up of the included studies was 1.5 years after the start of drug use. In order to draw a more definitive conclusion, a longer observation time is required.

### 4.7. SGLT2 Inhibitors—Gliflozins

The sodium-dependent glucose co-transporter 2 inhibitors (SGLT2i) are a novel oral glucose-lowering drug class that have shown the efficacy on glycemic control. These drugs are also called gliflozins. Due to their beneficial effects on cardiovascular risk and renal disease progression, SGLT2i are considered to be among the first-choice drugs in the treatment of DM, particularly for patients with heart failure [81]. Canagliflozin, dapagliflozin, empagliflozin, and ertugliflozin are inhibitors of SGLT2. These drugs have been registered for the treatment of DM by the Food and Drug Administration (FDA) and the European Medicines Agency (EMA) [82]. Other SGLT2i such as ipragliflozin, luseogliflozin, bexagliflozin, and tofogliflozin are only registered in certain countries. Gliflozins competently, reversibly, and selectively block the SGLT2 that is located in the proximal nephron tubules, where it leads to a 90% re-uptake of urinary glucose, which results in glycosuria, and as a result, the glucose plasma concentration is normalized in an insulin-independent mechanism [82]. Despite the usual mechanism of the inhibition of glucose uptake, these drugs also induce cell cycle arrest and apoptosis and also destabilize the mitochondrial membrane potential [83].

A recent meta-analysis showed that SGLT2 inhibitors were significantly associated with an overall reduced risk of cancer as compared to placebo (RR = 0.35, 95% CI: 0.33–0.37, *p* = 0. 00) with a particular effectiveness for dapagliflozin and ertugliflozin [84]. The expression of SGLT2 was detected in human breast cancer cell cultures as well as in human breast tumor tissue samples using both RT-PCR and immunohistochemistry [75,76]. In a study conducted by Zhou et al., it was observed that SGLT2 inhibitors (dapagliflozin and canagliflozin) manifested an anti-proliferative impact on breast cancer cells both in vitro and in vivo (utilizing nude mouse xenograft growth models). The study further revealed that SGLT2 inhibitors effectively arrested the cell cycle and induced cell apoptosis. Additionally, the research revealed that the treatment with SGLT2 inhibitors led to an augmentation in the phosphorylation of AMP-activated protein kinase (AMPK) and a reduction in the phosphorylation of 70 kDa ribosomal protein S6 kinase 1 (p70S6K1) within breast cancer cells [85].

Another study revealed that at 1–50 μM ipragliflozin significantly and dose-dependently suppressed the growth of human breast cancer MCF-7 cells. Such an effect was completely canceled by knocking down SGLT2, which suggests that ipragliflozin suppresses breast cancer by inhibiting SGLT2 [86]. There have also been reports about the potential usefulness of dapagliflozin in preventing cardiotoxicity in diabetic breast cancer patients who are receiving doxorubicin treatment. In animal studies, cardiac function was significantly preserved and cardiac fibrosis, apoptosis, and ER stress signaling were attenuated [87]. In a meta-analysis of randomized trials that assessed the effects of SGLT2i on the overall incidence of different types of cancer, the results of trials with a duration of at least one year were included. The included trials had enrolled 27,744 and 20,441 patients in the SGLT2 inhibitor and comparator groups, respectively [82]. In this meta-analysis, no difference was observed in the incidence of all of the malignancies between the patients that had been assigned to SGLT2i and comparators (OR = 0.98, 95% CI: 0.77–1.24). Among the 1659 cases of cancer, 107 (6.5%) were breast cancers, but in this study, a specific analysis was not done. One of the most recent meta-analyses on the effect of the SGLT2 inhibitors on the incidence of BCa was released by Spiazzi et al. [88]. This study included 98,939 adults with a minimum follow-up of 48 weeks. The authors concluded that the SGLT2 inhibitors likely resulted in little to no difference in the risk of breast cancer (RR = 1.01, 95% CI: 0.77–1.32; I2 = 0). The overall risk of bias was low.

According to Clinicaltrials.gov and Clinicaltrialsregister.eu, there are several clinical trials on antidiabetic drugs in breast cancer. The majority of these trials involve canagliflozin, dapagliflozin, and metformin as intervention therapies, often in conjunction with other standard chemotherapy treatments (Table 1). Due to the early stage of most of the above-mentioned trials, more time is necessary to draw a final conclusion. SGLT2i has been marketed relatively recently, and therefore, the duration of available observational studies is not sufficient for a reliable estimation of their association with cancer in the longer term.

## 5. Conclusions

New antidiabetic drugs have numerous positive effects, including enhanced cytotoxic activity on cancer cells. The increase in the risk of developing breast cancer among women with type 2 diabetes and diminished estrogen levels due to insulin resistance elevates the risk of cancer in estrogen receptor-rich organs such as the breasts, endometrium, and ovaries. Further research is crucial to elucidate the diverse mechanisms through which cytokines, IGF-1, mTOR, and AKT levels contribute to the increased breast cancer risk. Additionally, prospective studies are required to explore whether optimal glycemic control, coupled with adjustments to anti-hyperglycemic agents, can positively impact the prognosis for female breast cancer patients with diabetes. Moreover, investigating the efficacy of managing diabetes and treating cancer through a combination of antidiabetic and traditional anti-cancer drugs may offer a more potent approach to handling diabetes-associated cancers. SGLT2 inhibitors have additional cardioprotective effects, e.g., reducing the cardiotoxicity of doxorubicin. So far, many clinical trials have shown the beneficial effects of metformin on patients with BCa and diabetes. However, the addition of metformin to the standard BCa therapy in patients without DM demonstrated no benefits. Therefore, only for patients with DM and BCa, metformin should be recommended.

Obesity is a confounding variable for the association between diabetes and the risk of breast cancer. It should be noted that studies of the risk of breast cancer and obesity have generally observed an increased risk of breast cancer for overweight premenopausal women. The other observed problem is that most of the studies have not reported the BMI parameterization for an adjustment in their statistical model. Hence, an analysis that includes a variation of BMI and menopausal status should be carefully performed in women with DM and a diagnosis of BCa. Additionally, during the process of selecting the proper hypoglycemic therapy in BCa patients, obesity should always be considered. The observed actions of new antidiabetic drugs suggest two possible conclusions. Firstly, related to breast cancer prevention, should we modify recommendations to enable pharmacological prevention using GLP-1 analogs or SGLT2 inhibitors, especially in people who are overweight or have T2DM? The available data suggests that such a recommendation is at least neutral and safe. The use of antidiabetic agents promotes weight loss and provides better glycemic control, whereas it does not increase the risk of cancer. Contrarily, research studies show that it may reduce the risk of cancer. The second conclusion is to modify existing anticancer therapies to include GLP-1 agonists, DPP-4i, or SGLT2i, especially in a patient with coexisting diabetes or obesity. Taking into account that these synergistic effects also occur in people with normal body weight and glucose tolerance, it is possible to extend such recommendations to all patients treated for breast cancer. However, to properly address these questions, we need more clinical data from ongoing and future trials.

## Figures and Tables

**Table 1:** Current clinical trials of antidiabetic drugs in patients with breast cancer with unpublished results. ID of the trial according to clinicaltrials.gov (A) or clinicaltrialsregister.eu (B).

Trial ID	Drugs	Patient Population	Study Design	Target Enrollment
NCT05989347 (A)	Dapagliflozin	Patients with early stage HER2-negative breast cancer	Single group assignment, Open label study	20
NCT04073680 (A)	Canagliflozin, Serabelisib	Patients with confirmed locally advanced or metastatic solid tumors (including breast cancer)	Single group assignment, Open label study	60
NCT05090358 (A)	Canagliflozin, Alpelisib, Fulvestrant	Patients with metastatic HR-positive, HER2-negative breast cancer	Randomized, Open label study	106
NCT01980823 (A)	Metformin, Atorvastatin	Patients with operable invasive breast cancer with no prior chemotherapy, radiation therapy, or breast resection	Single group assignment, Open label study	23
2014-002602-20 (B)	Metformin, Liposomal Doxorubicin, Docetaxel, Trastuzumab	Patients with operable and locally advanced HER2 positive breast cancer	Single group assignment, Open label study	46
NCT04001725 (A)	Metformin, Dexamethasone	Patients with brain metastasis from melanoma, lung, or breast cancer, who require treatment with high-dose dexamethasone	Randomized, Open label study	110
2019-003093-13 (B)	Metformin	Patients with triple negative breast cancer	Randomized, Open label study	90
NCT05023967 (A)	Extended Release Metformin	Patients with luminal, operable, inflammatory breast cancer	Randomized, Open label study	120

## Data Availability

Data are contained within the article.
